# Psychiatryai.com: Real-Time AI Scoping Review (RAISR 4D) in Psychiatry and Mental Health with Live Real-World Evidence and CPD/CME for Psychiatrists

**DOI:** 10.1192/j.eurpsy.2024.392

**Published:** 2024-08-27

**Authors:** P. Naik, D. O’Leary

**Affiliations:** ^1^Evidence-Based Healthcare MSc Student, University of Oxford, Oxford; ^2^Child and Adolescent Psychiatry, Central and North West London NHS Foundation Trust, London; ^3^Medical Sciences Division, University of Oxford, Oxford, United Kingdom

## Abstract

**Introduction:**

Psychiatryai.com was launched in 2021 and initial findings were published at EPA 2023. The portal is an advanced computing science project in Applied Data Science and Evidence-Based Healthcare for my MSc studies at the University of Oxford (Kellogg College). Artificial Intelligence (AI) and Data Science (DS) technology are utilised to analyse live Real-World Evidence (RWE) in Psychiatry and Mental Health from PubMed to provide CPD/CME online. A two-year review of the site and its performance will be presented to EPA 2024.

**Objectives:**

To develop and study an experimental real-time AI and DS platform in Global Mental Health and Psychiatry, to provide the latest RWE from PubMed for online education and training, and to report findings to EPA 2024 for peer review in Budapest. AI and misinformation are newly identified risks in healthcare (AI Safety Summit 2023). The site also aims to raise awareness about “Aiatrogenesis” to address this problem, with RWE and CPD/CME utilising AI and DS technology for the categorisation and meta-analysis of evidence, rather than the production of possibly misleading or false Generative AI evidence (Monteith *et al.* BJP 2023; 1-3).

**Methods:**

As reported to EPA 2023 in Paris, a free open-code WordPress site was launched on the 22nd of November 2021 (Psychiatryai.com). The portal has been further developed and now features over 90k pages comprising 7GB of data with Cloudflare security and speed. Live evidence is collected into an open database and research articles are categorised into evidence nodes with AI. The results are presented in a real-time Evidence Matrix and Blueprint, creating 15-minute CPD/CME reflection modules. Data analytics from Psychiatryai.com with Google Analytics (G4A) along with platform insights from two years of development and research will be presented to EPA 2024. The site is conceptualised and designed to be viewed in an interactive VR headset.

**Results:**

**Table:** 

Live Citations	380000+
PubMed Articles Analysed with AI	92142
CPD/CME	23002 hours
Algorithms/Topics in Psychiatry	291
Open Data	7 GB

**Image:**

**Figure:**
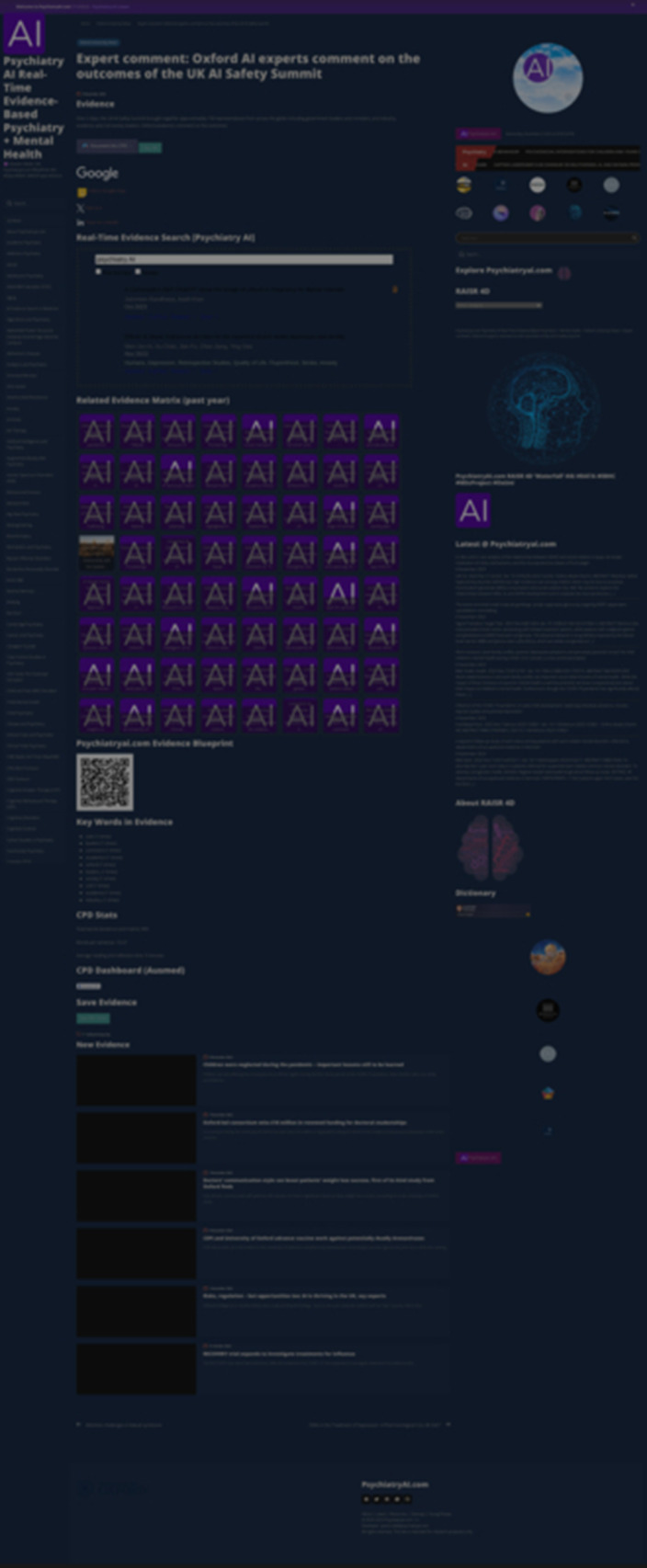

**Image 2:**

**Figure:**
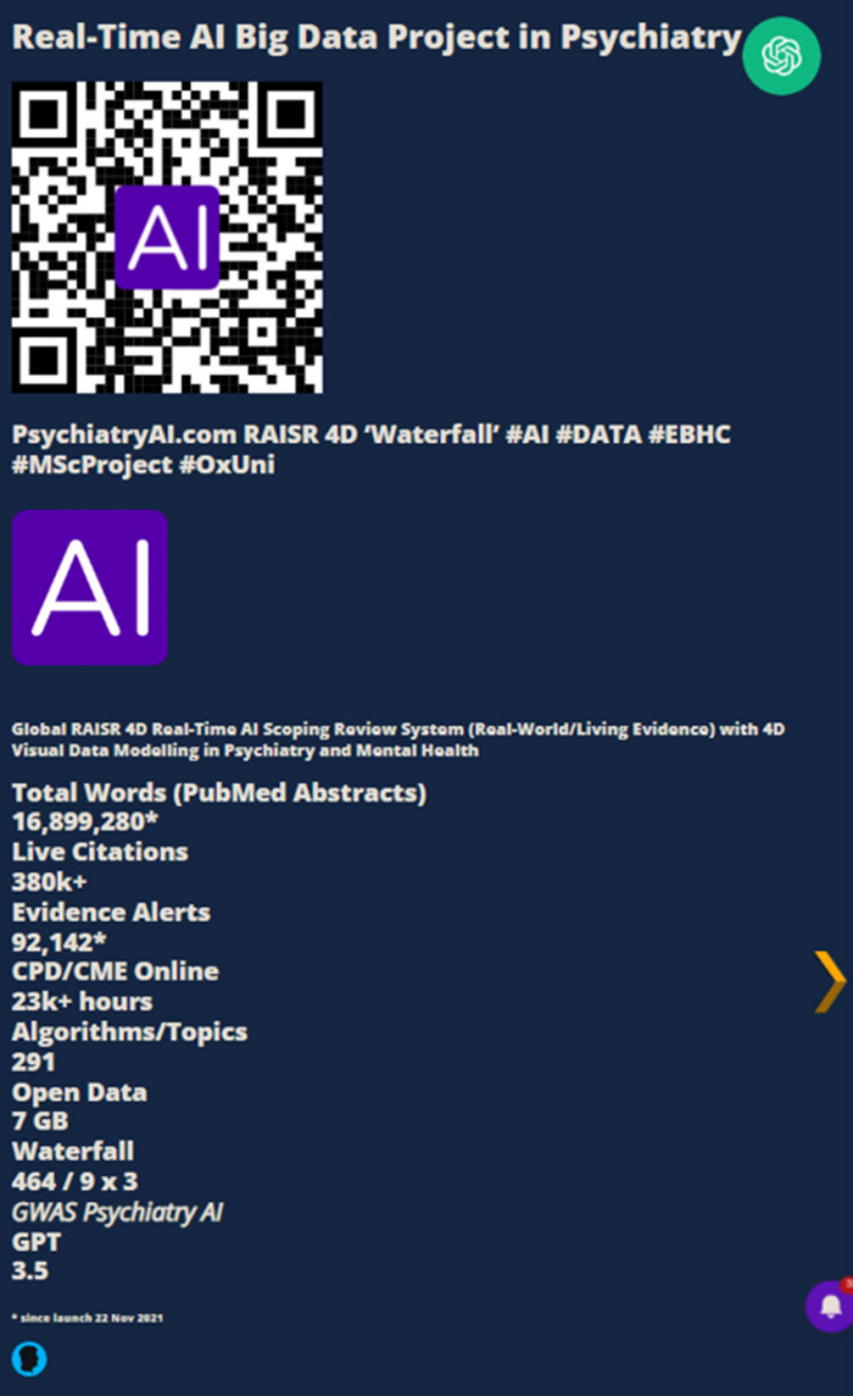

**Image 3:**

**Figure:**
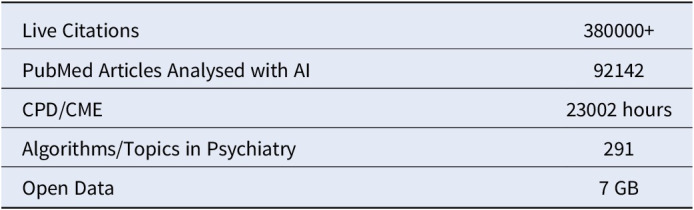

**Conclusions:**

Psychiatryai.com has successfully developed a novel AI and DS platform that incorporates the latest research in mental health and psychiatry, providing real-world evidence (RWE) for psychiatrists and healthcare professionals worldwide, along with CPD/CME online. This enhances hypothesis testing in research by presenting a related Evidence Matrix and Blueprint (from the last 365 days) for each evidence node on the site (RAISR 4D). These matrices provide a real-time visual table (8 x 8 / 64) of global research related to the evidence node in the preceding year. The site is VR-ready and has a special focus on AI and Psychiatry, Disaster and Traumatology Sciences, and Youth Mental Health. This project is dedicated to the memory of Dr Denis O’Leary and Dr Navin Venkatraman.

**Disclosure of Interest:**

None Declared

